# Haemorrhage of human foetal cortex associated with SARS-CoV-2 infection

**DOI:** 10.1093/brain/awac372

**Published:** 2023-01-16

**Authors:** Marco Massimo, Carlotta Barelli, Catalina Moreno, Chiara Collesi, Rebecca K Holloway, Berta Crespo, Lorena Zentilin, Anna Williams, Veronique E Miron, Mauro Giacca, Katherine R Long

**Affiliations:** Centre for Developmental Neurobiology, Institute of Psychiatry, Psychology and Neuroscience, King’s College London, London, UK; MRC Centre for Neurodevelopmental Disorders, King’s College London, London, UK; Centre for Developmental Neurobiology, Institute of Psychiatry, Psychology and Neuroscience, King’s College London, London, UK; MRC Centre for Neurodevelopmental Disorders, King’s College London, London, UK; Centre for Developmental Neurobiology, Institute of Psychiatry, Psychology and Neuroscience, King’s College London, London, UK; MRC Centre for Neurodevelopmental Disorders, King’s College London, London, UK; Molecular Medicine Laboratory, International Centre for Genetic Engineering and Biotechnology (ICGEB), 34139 Trieste, Italy; Department of Medical, Surgical and Health Sciences, University of Trieste, Trieste, Italy; Centre for Discovery Brain Sciences, Chancellor’s Building, The University of Edinburgh, Edinburgh, UK; Dementia Research Institute at The University of Edinburgh, Edinburgh, UK; Medical Research Council Centre for Reproductive Health, The Queen’s Medical Research Institute, The University of Edinburgh, Edinburgh, UK; Barlo Multiple Sclerosis Centre and Keenan Research Institute for Biomedical Science, St. Michael's Hospital, Toronto, Ontario, Canada; Department of Immunology, University of Toronto, Toronto, Ontario, Canada; Great Ormond Street Institute of Child Health, University College London, London, UK; Molecular Medicine Laboratory, International Centre for Genetic Engineering and Biotechnology (ICGEB), 34139 Trieste, Italy; Centre for Regenerative Medicine, Institute for Regeneration and Repair, The University of Edinburgh, Edinburgh BioQuarter, Edinburgh, UK; Centre for Discovery Brain Sciences, Chancellor’s Building, The University of Edinburgh, Edinburgh, UK; Dementia Research Institute at The University of Edinburgh, Edinburgh, UK; Medical Research Council Centre for Reproductive Health, The Queen’s Medical Research Institute, The University of Edinburgh, Edinburgh, UK; Barlo Multiple Sclerosis Centre and Keenan Research Institute for Biomedical Science, St. Michael's Hospital, Toronto, Ontario, Canada; Department of Immunology, University of Toronto, Toronto, Ontario, Canada; Molecular Medicine Laboratory, International Centre for Genetic Engineering and Biotechnology (ICGEB), 34139 Trieste, Italy; Department of Medical, Surgical and Health Sciences, University of Trieste, Trieste, Italy; British Heart Foundation Centre of Research Excellence, School of Cardiovascular Medicine & Sciences, King’s College London, London, UK; Centre for Developmental Neurobiology, Institute of Psychiatry, Psychology and Neuroscience, King’s College London, London, UK; MRC Centre for Neurodevelopmental Disorders, King’s College London, London, UK

**Keywords:** human foetal cortex, development, SARS-CoV-2, brain, haemorrhage

## Abstract

Maternal viral infection and immune response are known to increase the risk of altered development of the foetal brain. Given the ongoing global pandemic of coronavirus disease 2019 (COVID-19), investigating the impact of SARS-CoV-2 on foetal brain health is of critical importance. Here, we report the presence of SARS-CoV-2 in first and second trimester foetal brain tissue in association with cortical haemorrhages. SARS-CoV-2 spike protein was sparsely detected within progenitors and neurons of the cortex itself, but was abundant in the choroid plexus of haemorrhagic samples. SARS-CoV-2 was also sparsely detected in placenta, amnion and umbilical cord tissues. Cortical haemorrhages were linked to a reduction in blood vessel integrity and an increase in immune cell infiltration into the foetal brain. Our findings indicate that SARS-CoV-2 infection may affect the foetal brain during early gestation and highlight the need for further study of its impact on subsequent neurological development.

## Teaser

Massimo *et al.* report the presence of SARS-CoV-2 in first and second trimester foetal brain tissue in association with cortical haemorrhages. The haemorrhages were associated with reduced blood vessel integrity and increased immune cell infiltration into the foetal cortex.

## Introduction

Prenatal exposure to viral infection and maternal immune activation are associated with an increased risk of adverse neurodevelopmental outcomes in the offspring.^1–8^ Altered neurodevelopment has been linked to several viral infections,^9–12^ including the association of ZIKA virus infection with microcephaly in the recent 2015 epidemic.^13–17^ Coronavirus disease 2019 (COVID-19) has been linked to an increased risk of both premature and still birth,^18–21^ with increasing reports of vertical transmission between the mother and foetus.^21–29^ More rarely, some studies have also reported that maternal SARS-CoV-2 infection can lead to foetal demise, restricted growth or severe pathology, such as pneumonia and intraventricular haemorrhage.^19,30,31^ There are also an increasing number of studies that report a maternal immune response that may affect the foetus with subsequent effects on the neonate.^32–34^

Several studies have reported that SARS-CoV-2 can infect developing human neurons in cerebral organoids,^35–37^ with others reporting that SARS-CoV-2 infection is higher in the developing choroid plexus within these organoids.^38,39^ However, the effect of a maternal SARS-CoV-2 infection on foetal brain health has yet to be fully investigated and should be closely monitored.^34,40–45^ Here, we describe evidence of SARS-CoV-2 infection in early gestation foetal brain in association with cortical haemorrhages.

## Materials and methods

### Human tissue

Human foetal tissues were obtained from the Human Development Biology Resource (HDBR), provided by the Joint MRC/Wellcome Trust (grant #MR/R006237/1) (www.hdbr.org). The HDBR provided fresh tissue from foetuses aged 9–21 post-conception weeks (pcw). Of the 661 samples collected during the 21 month period, the majority were from elective terminations (no abnormality recorded), 62 of the samples were recorded to have a chromosomal trisomy (44 trisomy 21, 12 trisomy 18, 1 trisomy 16, 1 trisomy 13 and 3 triploid). Of the 26 haemorrhagic samples examined, 25 were from elective terminations (no abnormality recorded) and 1 was recorded to have trisomy 21. All tissue was fixed for at least 24 h at 4°C in 4% (wt/vol) paraformaldehyde (PFA) in 120 mM phosphate buffer (pH 7.4). COVID-19 patient lung tissue (Supplementary Fig. 8) was obtained as previously described in Bussani *et al*.^46^

### Immunofluorescence and imaging

Whole brains and whole mount sections were imaged using a Zeiss Axio Imager.Z1 and ApoTome I or a Cairn Custom designed dissection microscope. Brains were then sucrose-treated (15 and 30% sucrose solution sequentially for 24 h each), OCT-embedded, and then 20 μm thick sections were cut using a cryostat.

These sections were then stained in a solution containing 10% BSA and 0.1% triton, using the primary antibodies ACE-2 (1:50 R&D AF933), aquaporin-1 (1:100 Merck ab2219), CD3 (1:100 Bio-Rad MCA1477), CD20 (1:100 Abcam ab219329), CD68 (1:100 DAKO M0814), claudin-5 (1:100 Invitrogen 35-2500 (4C3C2)), cleaved caspase-3 (1:100 Cell Signalling Technology 9661), ERG (1:100 Abcam ab92513), Hopx (1:100 Santa Cruz sc-398703), HuC/D (1:100 Abcam ab184267), IBA-1 (1:100 Abcam ab5076), MAP2 (1:100 Millipore ab5622), Mouse IgG isotype control (1:100 Sigma M5284), nestin (1:100, Sigma N5413), pan-laminin (1:100 Sigma L9393), Rabbit IgG isotype control (1:50 Abcam ab172730 [EPR25A]), SARS-CoV-2 spike protein (1:100-250 Genentech GTX632604 [1A9]), SARS-CoV-2 nucleocapsid protein (1:50-150 Sino Biological 40143-R001), S100a9 (1:200 BMA biomedicals T-1026), Sox2 (1:100 R&D Systems AF2018).

The Life technologies secondary antibodies, used at 1:1000, were donkey-anti-goat Alexa fluor 488 (A11055), anti-mouse Alexa fluor 568 (A10037) and 555 (A31570), anti-rabbit Alexa fluor 647 (A31573) and 488 (A21206), and goat anti-rat 555 (A21434) and anti-mouse 594 (A11005). Sections were all stained with DAPI (Sigma D9542) and mounted in Mowiol (Merck Biosciences).

Sections were then imaged using either a Zeiss LSM 800 inverted confocal microscope and a Zeiss Plan-Apochromat 20 × 0.8 objective, or a Zeiss AxioScan slide scanner and a Zeiss Plan-Apochromat 20 × 0.8 M27 objective.

### *In situ* hybridization and imaging

*In situ* hybridization was performed using locked nucleic acid (LNA) probes for U6 snRNA (miRCURY LNA Detection probe, Qiagen, Cat. No. YD00699002) and SARS-CoV-2 RNA, designed to target the sense strand of ORF1ab and Spike regions of the viral genome. A scrambled sequence probe (YD00699004) was used as a control.

Experiments were performed using a dedicated ISH (Qiagen) according to the manufacturer’s protocol. Briefly, air-dried frozen tissue sections were incubated with either SARS-CoV-2 (40 nM) or U6 probes (2 nM) for 1 h at 54°C in a hybridizer. After washing with SSC buffer, the presence of SARS-CoV-2 RNA was detected using an anti-DIG alkaline phosphatase (AP) antibody (1:500) (Roche Diagnostics) supplemented with sheep serum (Jackson Immunoresearch) and bovine serum albumin (BSA). Hybridization was detected by adding NBT-BCIP substrate (Roche Diagnostics). Nuclei were counterstained with nuclear fast red. Images were acquired using a Leica ICC50W light microscope.

### Immunohistochemical staining and imaging

Immunohistochemical staining was performed using a standard protocol. Briefly, sections were allowed to dry and were permeabilized for 10 min in 1% Triton X-100 in PBS, followed by blocking in 2% BSA (Roche) and overnight staining at 4°C with the primary antibodies diluted in blocking solution. After endogenous peroxidase inhibition with 3% H_2_O_2_, sections were incubated with appropriate biotin-conjugate secondary antibody for 1 h at room temperature. Following signal amplification with avidin–biotin-complex–HRP (VECTASTAIN), DAB solution (VECTOR) was applied for 2–3 min. Haematoxylin (Bioptica) was further used to stain nuclei. Haematoxylin and 1% eosin were used for the H&E staining using a standard protocol.

Prussian blue staining was performed using equal parts of hydrochloric acid and potassium ferrocyanide prepared immediately prior to use, which was added to the slides for 20 min. Slides were then washed with distilled water three times and mounted in Mowiol.

### Image analysis and quantification

All image analyses and quantifications were conducted using Fiji, and all statistical analyses were conducted using GraphPad Prism. Integrated density measurements were conducted using a specified region of interest taken at least three times within the required area and then averaged. A branched blood vessel was classified as any vessel with one branch or more. All datasets were tested for Gaussian distribution (Kolmogorov–Smirnov test) prior to the selection of the appropriate statistical test. All datasets were analysed blind. For all graphs, error bars represent either ± standard deviation or standard error of means as stated in the figure legend. Differences between groups were analysed using a two-tailed Student’s *t*-test or Mann–Whitney test.

### Data availability

There are no restrictions on data availability. All data are reported in the article or the Supplementary material.

## Results

### Incidence of haemorrhages in human foetal cortex during the COVID-19 pandemic

**Figure 1:**
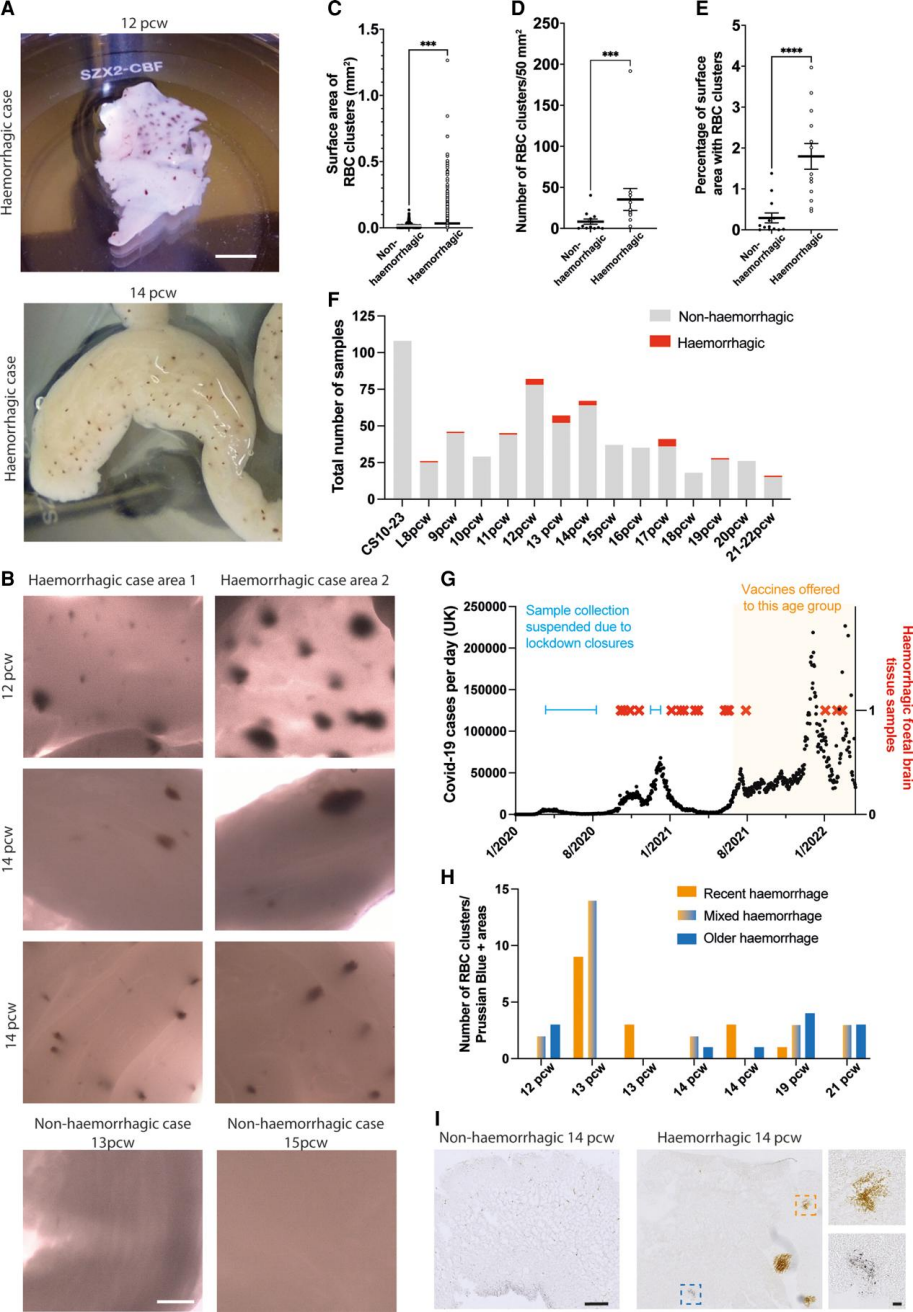
**Characterization of haemorrhages in human foetal cortex tissue.** (**A**) Whole mount images of human foetal cortex tissue displaying RBC clusters in haemorrhagic tissue collected during the COVID-19 pandemic. (**B**) Whole mount images of human foetal cortex tissue from one 12 pcw and two 14 pcw foetuses displaying large clusters of red blood cells (RBC) (*upper and middle rows*) and cortex tissue from a 13 pcw and 15 pcw foetuses without RBC clusters. (**C**) Quantification of the surface area of individual RBC clusters (*n* = 2236) in non-haemorrhagic samples (no RBC clusters with a surface area above 0.4 mm^2^, or less than 20 RBC clusters between 0.1–0.4 mm^2^/mm^2^ of tissue) and haemorrhagic samples. (**D**) Quantification of the number of RBC clusters per 50 mm^2^ of tissue. (**E**) Quantification of the percentage of tissue surface area that contains RBC clusters. (**F**) The total number of foetal cortex samples collected at each age by the HDBR from the receival of the first haemorrhagic sample (5 Oct 2020) until 14 April 2022 (grey bars, *lower area of bar*) and the number of haemorrhagic samples (red, *upper area of bar*). Samples younger than 8 pcw are identified by Carnegie stages (CS). (**G**) Graph showing the number of confirmed COVID-19 cases per day in the UK from 30 January 2020 up to 14 April 2022 (black filled circles), the haemorrhagic foetal cortex samples (red crosses), the times when sample collection was suspended due to lockdown-related closures of labs (blue lines), and the period from which women under 40 were offered a COVID-19 vaccine (yellow shaded area to *right*). (**H**) Quantification of the number of RBC clusters/prussian blue positive areas in seven haemorrhagic samples. For each age shown, recent bleeds (orange, *left bars*) contained RBCs only, mixed bleeds (orange/blue, *middle bars*) contained both RBCs and Prussian blue deposits and older bleeds (blue, *right bars*) contained only Prussian blue deposits. Samples are ordered on the *x*-axis according their age in pcw. (**I**) Prussian blue staining in a 14 pcw non-haemorrhagic (*left*) and 14 pcw haemorrhagic (*right*) cortex. Top dashed box delineates a bleed containing RBCs (orange colour, *adjacent upper right image*) and the lower dashed box delineates an area positive for Prussian blue (blue colour, *adjacent lower right image*). Scale bars: (**A**) 1 cm, (**B**) 1 mm, (**I**) 500 µm, (**I**, *inset*) 50 µm. Statistics: (**C**–**E**) Mann–Whitney test, ***P = 0.0007 (**C**), 0.0008 (**D**), ****P < 0.0001. Error bars = standard deviation. Non-haemorrhagic samples *n* = 13, haemorrhagic *n* = 13.

In the 21 months from the start of July 2020 to the middle of April 2022, 661 human foetal tissue samples were collected by the HDBR in the UK. This period covers the time in which foetal tissue collection occurred during the COVID-19 pandemic, as collections were suspended from the end of March 2020 until July 2020. On gross examination of these samples, we observed haemorrhages within the cortical tissue in 26 of these 661 foetal tissue samples (Fig. 1A and B and Supplementary Fig. 1A). Whilst haemorrhages may occasionally occur (Supplementary Fig. 1B and C), they are very rare, with only two cases of similar haemorrhages in cortical tissue observed from images of 300 randomly selected samples from a total of 4917 that were collected by the HDBR between September 1999 to December 2019. It is therefore highly unusual to see this quantity of samples containing this degree of haemorrhage within the last 21 month period.

We examined the haemorrhages by quantifying the size and number of clusters of red blood cells (RBC) observed in the tissue, in 26 of the 661 human foetal cortex tissue samples collected during the COVID-19 pandemic (including 13/26 of the haemorrhagic samples above, 11–21 pcw (Fig. 1A–F and Supplementary Fig. 1A). Small RBC clusters can occasionally be observed within blood vessels in the cortical tissue (Fig. 1B–E and Supplementary Fig. 1A), all of which had a low surface area. To differentiate these RBC clusters in blood vessels from haemorrhages, we characterized haemorrhages as RBC clusters with either a surface area >0.4 mm^2^ or an area with over 20 RBC clusters 0.1–0.4 mm^2^ per mm^2^ of tissue (13/26 samples, 11–21 pcw; Fig. 1C).

Quantification of RBC clusters within the 26 cortical tissue samples revealed that the average surface area of these clusters was 0.01689 mm^2^ in non-haemorrhagic samples and 0.04531 mm^2^ in haemorrhagic samples, with the largest being 1.265 mm^2^ (Fig. 1C). Compared to non-haemorrhagic samples, haemorrhagic samples contained RBC clusters of a significantly larger surface area (Fig. 1C), a significantly higher number of RBC clusters per mm^2^ of cortical tissue (Fig. 1D), and a significantly higher percentage of cortical tissue surface area that contained RBC clusters (Fig. 1E).

### Timing of the haemorrhages in human foetal cortex tissue

As the number of these haemorrhagic samples received during the pandemic was unexpected, we next examined the number of haemorrhagic samples in relation to confirmed COVID-19 cases in the UK (data from https://coronavirus.data.gov.uk/; Fig. 1G and Supplementary Fig. 2A and B). Haemorrhagic samples were received during periods of time when confirmed COVID-19 cases were highest (Fig. 1G and Supplementary Fig. 2A and B). The stage of gestation of these haemorrhagic samples varied from 8 pcw to 22 pcw (Fig. 1F), the upper age limit of tissue samples collected. However, the majority of the haemorrhagic samples were between 12–14 pcw (57.6%, 15/26; Fig. 1F).

To determine whether the haemorrhages were acute or more chronic, we examined the presence of ferric iron deposits, which arise after the lysis of RBCs, using Prussian Blue staining (Fig. 1H and I). Some samples showed RBCs, identifiable as orange cells within the tissue (Fig. 1I), found in areas with no Prussian Blue staining, indicating a recent haemorrhage. Other samples showed Prussian Blue stained areas absent of RBCs, indicating an older haemorrhage, whilst other samples showed a mixed pattern of Prussian Blue staining and RBCs, likely representing an intermediate time point (Fig. 1H and I). Strikingly, haemorrhagic samples that contained more RBC clusters without Prussian Blue staining (recent haemorrhage) contained the lowest number of areas of Prussian Blue alone (older haemorrhage, Fig. 1H), suggesting that these samples may represent different time points from the appearance of the haemorrhage. The more recent haemorrhages were found more often in the younger samples (12–14 pcw) than in the older samples (19–21 pcw). The different ages of haemorrhages present within each sample also indicate that they are not a result of the collection procedure performed.

We next examined the number of cleaved caspase-3 positive cells (Supplementary Fig. 3A and B) to determine if either the more recent or older haemorrhages were associated with an increase in cell death within the cortical tissue. No significant increase in cleaved caspase-3 positive cells were found in either the samples containing more recent or older haemorrhages (Supplementary Fig. 3B) compared to the non-haemorrhagic samples. In line with this finding, the thickness of the cortical wall was also not significantly altered in the haemorrhagic samples compared to the non-haemorrhagic samples (Supplementary Fig. 3C).

### Presence of SARS-CoV-2 in human foetal brain tissue

**Figure 2:**
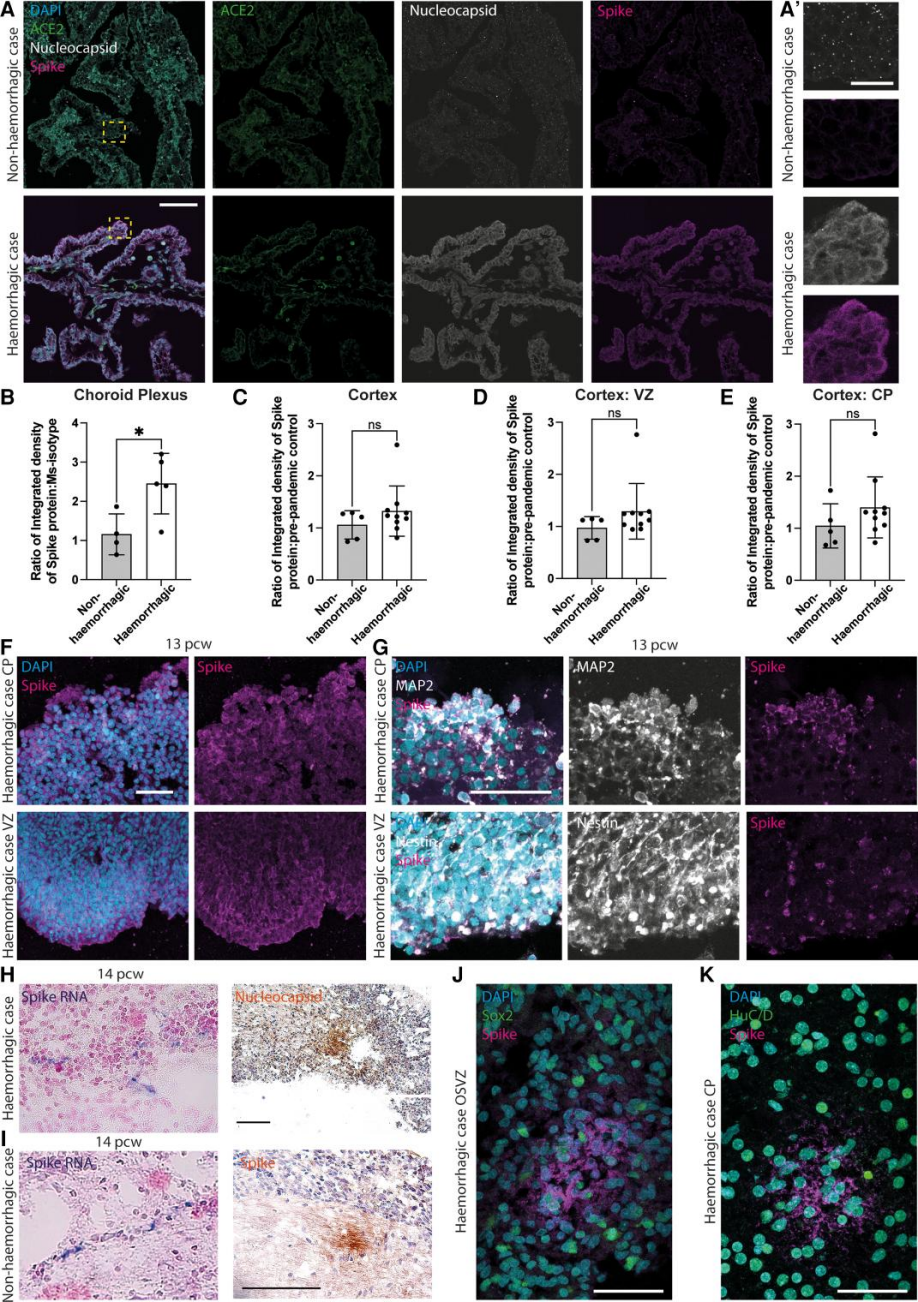
**SARS-CoV-2 detection in human foetal cortex and choroid plexus.** (**A**) Immunofluorescence of choroid plexus from foetuses with non-haemorrhagic (*upper*) and haemorrhagic (*lower*) cortex, for ACE2 (green, *middle left*), SARS-CoV-2 nucleocapsid (grey, *middle right*) and SARS-CoV-2 spike protein (magenta, *right*) and their merge with DAPI (blue, merged image *left*). Yellow boxes delineate the area shown on the *far right* (**A′**). (**B**–**E**) Quantification of the ratio of integrated density of immunofluorescence of the spike protein relative to its isotype control in (**B**) the choroid plexus from foetuses with non-haemorrhagic (*n* = 4) and haemorrhagic (*n* = 5) cortices, (**C**) non-haemorrhagic (*n* = 5) and haemorrhagic (*n* = 10) cortex, and (**D**) the ventricular zone (VZ) and (**E**) the cortical plate (CP) of these cortices. (**F**–**G**) Immunofluorescence of the cortical plate (CP, upper) and ventricular zone (VZ, lower) of haemorrhagic cortex for (**F**) DAPI (blue, merged image *left*) and spike protein (magenta, *right*) in a 13 pcw foetus, and (**G**) DAPI (blue, merged image *left*), MAP2 (grey, neuronal marker, *upper middle*) or Nestin (neural progenitor marker, *lower middle*) and spike protein (magenta, right) in another haemorrhagic 13 pcw foetus. (**H**) ISH for SARS-CoV-2 spike RNA (*left*) and immunohistochemistry for nucleocapsid protein (*right*) in a haemorrhagic sample. (**I**) ISH for SARS-CoV-2 spike RNA (*left*) and immunohistochemistry for spike protein (*right*) in a non-haemorrhagic sample. (**J** and **K**) Immunofluorescence of the outer subventricular zone (OSVZ, **J**) and cortical plate (CP, **K**) for DAPI (blue), SARS-CoV-2 spike protein (magenta) and either Sox2 (green, **J**) or HuC/D (green, **K**). Scale bars: (**A**, **H** and **I**) 100 µm, (**A′**) 25 µm, (**F**, **G**, **J** and **K**) 50 µm. Statistics: (**B** and **E**) unpaired *t*-test, (**C** and **D**) Mann–Whitney test, *P = 0.0247. Error bars = standard deviation.

It has previously been reported that SARS-CoV-2 can infect both neuronal/cortical tissue and the choroid plexus in human cerebral organoids.^35–39^ Choroid plexus tissue was reported to be particularly vulnerable to SARS-CoV-2 infection in these organoids due to a high level of expression of ACE2, a major receptor for SARS-CoV-2.^38,39^ We therefore examined choroid plexus tissue by immunofluorescence for both the SARS-CoV-2 spike protein and nucleocapsid (N) protein, which binds the viral RNA genome (Fig. 2A and B and Supplementary Fig. 4).

Of the 26 haemorrhagic cortex tissue samples collected, five also contained choroid plexus tissue. These were analysed alongside four age-matched non-haemorrhagic cases. Consistent with previous reports, we detected a high level of ACE2 expression in the choroid plexus epithelium (Fig. 2A and Supplementary Fig. 4A–C). We also detected a significant level of SARS-CoV-2 spike and nucleocapsid proteins in all five of the choroid plexuses from haemorrhagic samples (average of 2.5 times above background staining, Fig. 2A and B), in comparison to non-haemorrhagic samples (Fig. 2A and B), in aquaporin-1 positive cells (Supplementary Fig. 4D). Notably, we also detected a low level of SARS-CoV-2 spike protein in one of the non-haemorrhagic choroid plexus samples (Fig. 2B and Supplementary Fig. 4B).

We next examined the level of SARS-CoV-2 in the cortex tissue of the five samples where choroid plexus was obtained, plus an additional five haemorrhagic cortex samples, alongside age-matched controls. In contrast to the choroid plexus, we detected relatively little SARS-CoV-2 spike protein immunofluorescence in the cortical tissue (average of 1.3 times above background; Fig. 2C–G and Supplementary Figs 5–7), with no significant difference observed between non-haemorrhagic or haemorrhagic samples relative to pre-pandemic age-matched controls (Fig. 2C). Notably, the samples with the highest levels of SARS-CoV-2 spike protein immunofluorescence were 12–14 pcw in age.

We also identified a small, focal area of strong SARS-CoV-2 spike protein staining within the cortex in seven of the haemorrhagic samples by immunofluorescence (Fig. 2D–G, J and K and Supplementary Figs 6 and 7), and low levels in one non-haemorrhagic sample (Supplementary Fig. 5B). Within these samples, SARS-CoV-2 spike protein was detected within the neurons of the cortical plate (MAP2 positive, Fig. 2E–G, HuC/D positive Fig. 2K and Supplementary Fig. 7C), and within the progenitors of the ventricular and subventricular zones (nestin positive radial glia, Fig. 2D, F and G and Supplementary Fig. 6B; Sox2 positive progenitors Fig. 2J and Supplementary Fig. 7D and E; Hopx positive basal radial glia Supplementary Fig. 7E). Spike and nucleocapsid protein could also be detected within discrete areas of the cortex in 5 of the 10 haemorrhagic samples examined by immunohistochemistry (Fig. 2I, right panel, and Supplementary Fig. 8B), and in the cortex of the same non-haemorrhagic sample as above (Supplementary Fig. 8A, same sample as Supplementary Fig. 5B).

Consistent with these results, we detected low levels of SARS-CoV-2 spike protein RNA by *in-situ* hybridization (ISH) in discrete areas in four of the haemorrhagic samples (of eight examined) within the cortical plate and subplate (Fig. 2H and I, left panels, and Supplementary Fig. 8E) but did not detect significant levels throughout the cortical tissue. Overall, in the 10 haemorrhagic samples examined, SARS-CoV-2 was detected either within the choroid plexus, cortex or both. It was also detected within the choroid plexus of one non-haemorrhagic sample and in the cortex of another. These data indicate the association of SARS-CoV-2 infection with haemorrhagic samples, but that not all infected samples contain haemorrhages.

### Presence of SARS-CoV-2 in other human foetal tissues

It has previously been reported that SARS-CoV-2 can infect placenta^17,26–28,30,31,47–51^ and other foetal tissues, such as the umbilical cord.^28^ We therefore examined various foetal tissues and placenta collected from three samples with observed cortical haemorrhages. SARS-CoV-2 spike protein was detected by immunofluorescence in the cortex and sparsely in the placenta of an 11 pcw sample (Supplementary Fig. 9A), in the cortex and umbilical cord of a 13 pcw sample (Supplementary Fig. 9B) and in the cortex, amnion, umbilical cord, but not the placenta, in a 9 pcw sample (Supplementary Fig. 9C), indicating the presence of SARS-CoV-2 in tissues with both foetal and maternal origin.

### Reduced integrity of endothelial cells within the blood vessels in haemorrhagic human foetal cortex tissue

Haemorrhagic cerebral injury can be associated with changes to blood vessel integrity.^52^ One method to examine this is to quantify the tight junctions between the endothelial cells of the blood vessels, which enable the blood vessels to create a tight barrier to prevent leakage into the surrounding tissues. These tight junctions increase over the course of foetal brain development as the blood brain barrier develops.^52–54^

**Figure 3:**
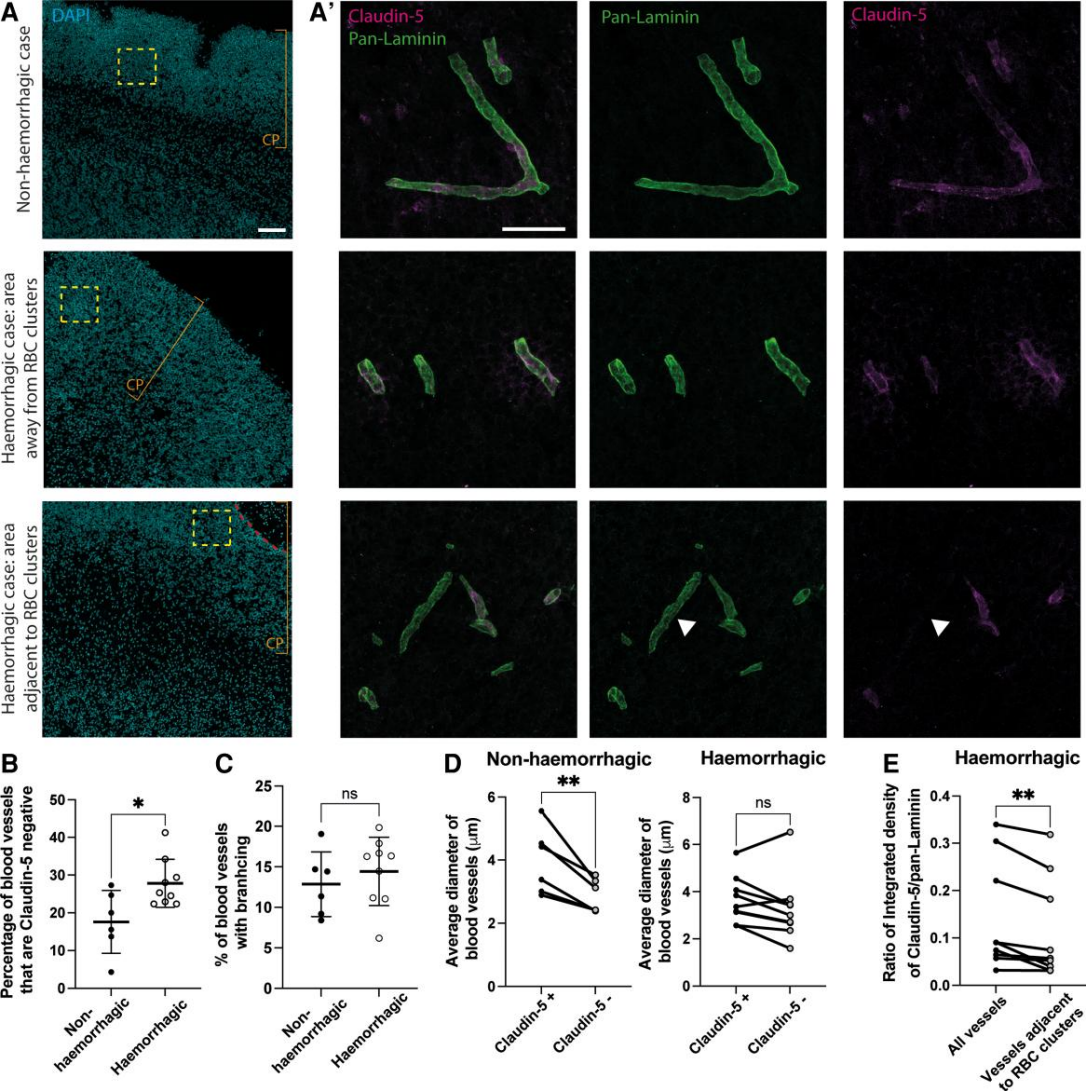
**Blood vessel integrity in human foetal cortex tissue.** (**A**) Immunofluorescence of cortex from non-haemorrhagic (*upper*) and haemorrhagic (away from RBC clusters, *middle*, and adjacent to RBC clusters, *lower*) samples, for DAPI (blue, *left*), pan-laminin (blood vessel basement membrane, green, middle images) and claudin-5 (endothelial cell tight junctions, magenta, *right*). Dashed boxes delineate areas on the *right*, dashed line indicates area of RBC clusters, arrow heads show pan-laminin positive blood vessel that is claudin-5 negative, bars indicate area of cortical plate (CP). (**B**–**D**) Quantification of the percentage of blood vessels that are claudin-5 negative throughout the cortical wall (**B**), the percentage of blood vessels with branching (**C**), the average diameter of claudin-5 positive and negative blood vessels in the non-haemorrhagic and haemorrhagic cortex (**D**) and the ratio of claudin-5 to pan-laminin integrated density in all blood vessels in haemorrhagic cortex and vessels adjacent to RBC clusters (**E**). Scale bars: (**A**) 100 µm, (**A′**) 50 µm. Statistics: Unpaired *t*-test (**B** and **C**), paired *t*-test (**D** and **E**). ** *P* = 0.0088 (**D**) 0.0054 (**E**), *P = 0.0184 (**B**), ns = 0.9421 (**C**), 0.1076 (**D**), Error bars = standard deviation. Non-haemorrhagic (*n* = 6) 13–19 pcw, haemorrhagic (*n* = 9) 12–19 pcw (all SARS-CoV-2 spike protein positive).

We therefore performed immunofluorescence for the endothelial tight junction protein claudin-5 and identified blood vessels using the vascular basement membrane marker laminin (pan-laminin, Fig. 3A). The percentage of blood vessels that were claudin-5 negative was significantly increased in the cortex of the haemorrhagic samples in comparison to the aged matched, non-haemorrhagic samples (Fig. 3B), although the percentage of these vessels with branching remained unchanged (Fig. 3C). The diameter of these claudin-5 negative vessels was significantly smaller than the claudin-5 positive vessels in the cortex of non-haemorrhagic samples (Fig. 3D, left), but larger diameter vessels were also found to be claudin-5 negative in the cortex of haemorrhagic samples (Fig. 3D, right). Within the cortex of the haemorrhagic samples, vessels adjacent to a site of RBC clusters had significantly less claudin-5 than those away from the site of RBC clusters (Fig. 3A and E). Overall, these data indicate a reduction in blood vessel integrity in the haemorrhagic samples.

### Immune cell infiltration in haemorrhagic human foetal cortex tissue

**Figure 4:**
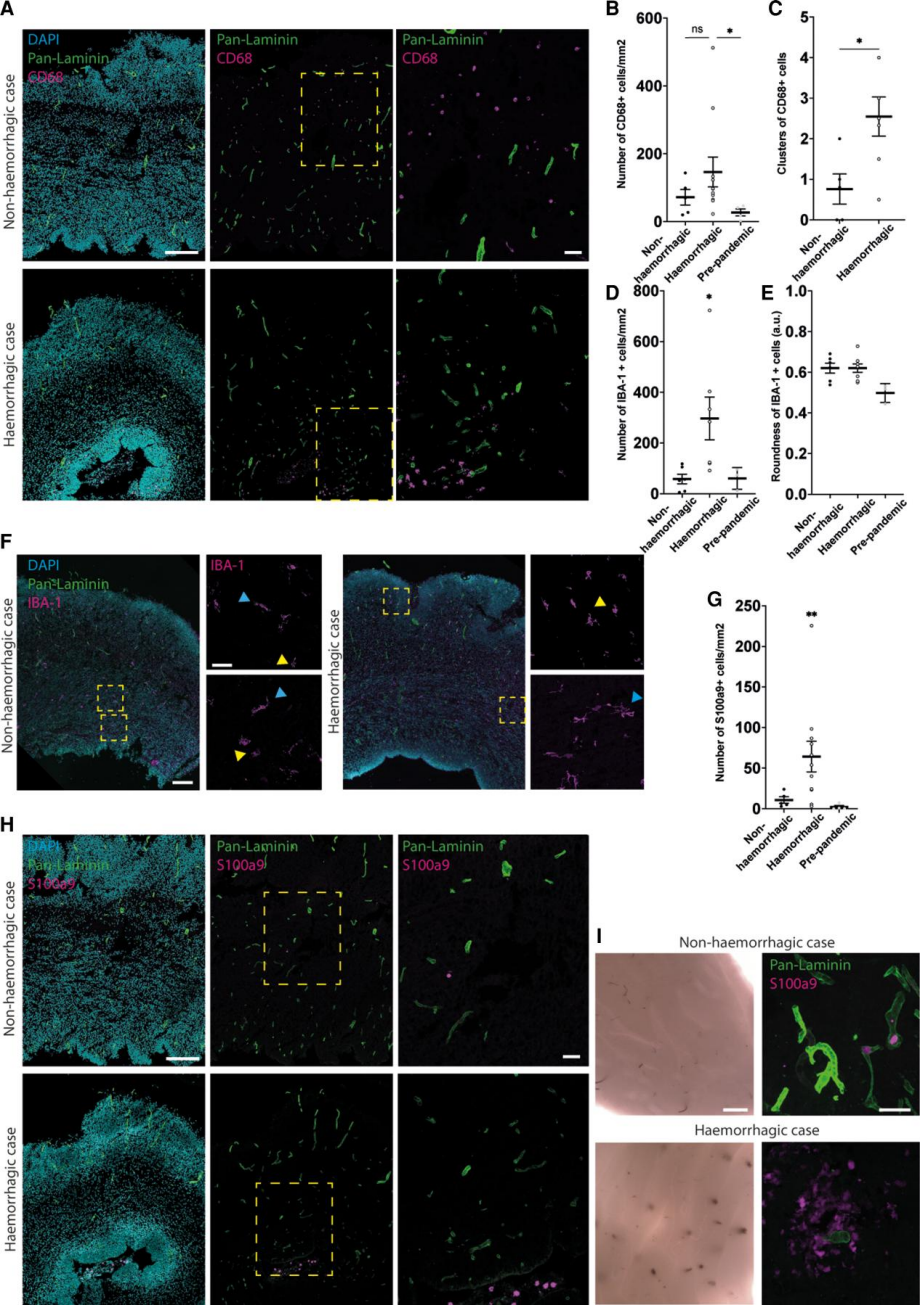
**Immune cell infiltration in human foetal cortex tissue.** (**A**) Immunofluorescence of cortex from non-haemorrhagic (*upper*) and haemorrhagic (*lower*) foetuses for DAPI (blue, *left*), pan-laminin (blood vessel basement membrane, green, all images) and CD68 (phagocytosing macrophages, magenta, all images). Dashed boxes delineates areas to the right. (**B** and **C**) Quantification of the number of CD68 positive cells per mm^2^ of tissue (**B**), and the number of clusters of CD68 positive cells (**C**) in non-haemorrhagic (*n* = 4) and haemorrhagic (*n* = 9) cortex, and (**B**) pre-pandemic cortex (*n* = 4). (**D** and **E**) Quantification of the number per mm^2^ of tissue (**D**) and roundness (**E**) of IBA-1 positive cells in non-haemorrhagic (*n* = 6), haemorrhagic (*n* = 7) and pre-pandemic cortex (*n* = 3). (**F**) Immunofluorescence of cortex from non-haemorrhagic (*left*) and haemorrhagic (*right*) for DAPI (blue, merged larger image), pan-laminin (blood vessel basement membrane, green, merged larger image) and IBA-1 (macrophages, magenta merged larger image and upper and lower smaller images). Dashed boxes delineates areas to the right, yellow arrowheads mark more rounded IBA-1 + cells, blue arrowheads mark less round IBA-1 + cells. (**G**) Quantification of the number of S100a9 positive cells per mm^2^ of tissue in non-haemorrhagic (*n* = 4), haemorrhagic cortex (*n* = 9) and pre-pandemic cortex (*n* = 4). (**H**) Immunofluorescence of cortex from non-haemorrhagic (*upper*) and haemorrhagic (*lower*) foetuses for DAPI (blue, *left*), pan-laminin (blood vessel basement membrane, green, all images) and S100a9 (monocytes, magenta, all images). Dashed boxes delineates areas to the right. (**I**) Whole mount images of human foetal cortex tissue and immunofluorescence for pan-Laminin (green) and S100a9 (magenta) in non-haemorrhagic (*upper*) and haemorrhagic (*lower*) cortex. Scale bars: (**A**, **F**, **H** and **I**) 200 µm, (**A** and **H**, *inset*) 100 µm, (**F** and **I**, *inset*) 50 µm. Statistics: Mann–Whitney test (**B** and **G**), unpaired *t*-test (**C**). *P = 0.0275 (**B**), 0.0213 (**C**), 0.0393 (**D**), **P = 0.009 (**G**). Error bars = standard error of the mean.

Maternal viral infection can cause altered immune responses in the foetal brain, including increased brain macrophages which can be derived from resident macrophages and infiltrating blood monocytes. Of note, tissue macrophages and monocytes have been linked to pathology in adults infected with COVID-19.^55^ We found no significant difference in the number of brain macrophages (CD68 positive) in the cortex of haemorrhagic versus non-haemorrhagic samples (Fig. 4A and B), with only a small difference when compared to pre-pandemic controls (Fig. 4B and Supplementary Fig 10).

However, we did detect a change in the distribution of CD68 positive cells within the cortical tissue, with a significant increase in the number of clusters of CD68 positive cells in the cortex of haemorrhagic samples (Fig. 4C and Supplementary Fig. 10F and G). A cluster of CD68 positive cells was defined as more than 10 CD68 positive cells within an area of 2900 µm^2^, with the largest cluster 449 353 µm2 with 402 CD68 positive cells. 38% of these clusters were found to overlap with a cluster of RBCs (Supplementary Fig. 10F and G), consistent with a potential macrophage reaction to the haemorrhages.

We also found a significant increase in the number of IBA-1 positive brain macrophages in the cortex of the haemorrhagic samples (Fig. 4D–F). These IBA-1 positive cells displayed a wide variety of morphologies, from rounded to more complex (Fig. 4F). However, IBA-1 positive cells of these varied morphologies were found in both the haemorrhagic and non-haemorrhagic samples, resulting in no significant difference in the roundness of the cells (Fig. 4E). This is consistent with the lack of a significant increase in CD68 positive cells above (Fig. 4B), as the IBA-1 positive cells with a more rounded morphology are often associated with CD68 positivity and phagocytosis.

In addition, a significant increase in monocytes (positive for the marker s100a9) was detected in the cortex of haemorrhagic samples versus non-haemorrhagic and pre-pandemic samples (Fig. 4G–I and Supplementary Fig. 10). Whereas s100a9 positive cells were located within blood vessels of the cortex of non-haemorrhagic samples, (Fig. 4I, upper panels), these were found within the parenchyma of the cortex of haemorrhagic samples (Fig. 4I, lower panels), indicating extravasation into tissue. To determine if more immune cells were present, immunofluorescence was performed for the lymphocyte markers CD3 and CD20 (Supplementary Fig. 1C–E). No CD3 positive cells were found in the cortex and only one CD20 positive cell was found within a blood vessel of a non-haemorrhagic sample, indicating that lymphocyte infiltration was not present. Taken together, these data are consistent with the presence of a macrophage and monocyte immune response within the cortex of haemorrhagic samples.

## Discussion

Here we report SARS-CoV-2 infection in human foetal brain in association with haemorrhage, disrupted endothelial integrity and infiltration of immune cells in the developing cortex. This is a timely and important finding as there have been conflicting reports of vertical transmission of SARS-CoV-2 from mother to foetus and limited data on the effect of SARS-CoV-2 infection on foetal brain.^18,19,21–24,26,34,42^ SARS-CoV-2 was primarily detected in the choroid plexus of haemorrhagic samples, and more sparsely in the cortical neurons and progenitors, consistent with previous findings of increased vulnerability of the choroid plexus to infection in developing organoids.^38,39^

SARS-CoV-2 was detected in all of the samples presenting with haemorrhages examined, but whether these haemorrhages are a direct consequence of the infection or an indirect consequence of the maternal immune response^1,32–34^ to SARS-CoV-2 remains to be elucidated. It was also detected in tissue samples from three haemorrhagic samples in the umbilical cord and amnion, as well as sparsely in the placenta. In addition to the haemorrhagic samples, we also detected SARS-CoV-2 spike protein at a lower level in the choroid plexus of one sample and the cortex of another sample that did not display any sign of haemorrhage, nor any reduction in claudin-5 or increase in CD68 or S100a9 positive cells. This may indicate that these samples may either be at an early stage of SARS-CoV-2 infection prior to haemorrhage, a stage post-infection where the haemorrhage has subsided, or that infection with SARS-CoV-2 was not associated with haemorrhage at all in this sample. Further work examining foetal material collected during the ongoing COVID-19 pandemic will be important to monitor the number of maternal SARS-CoV-2 infections that may result in such haemorrhages, including in mothers that are asymptomatic.

Strikingly, the haemorrhages are predominately found in the late first and early second trimester of gestation, a period of development in which the effect of the COVID-19 pandemic has not been thoroughly investigated. Specifically, the majority were between 12 and 14 pcw, a critical window of human foetal brain development when the endothelial tight junctions increase to form the blood brain barrier.^52–54^ Our observations of disrupted foetal cerebral vasculature are consistent with reports of damage to the microvasculature of the adult brain in SARS-CoV-2 infected patients.^56–59^ Further investigation is needed to understand if these effects on the cortical tissue are long lasting, or are able to resolve with minimal consequence. It is possible that an immune cell response could have a positive outcome, resulting in some resolution of these haemorrhages. However, maternal immune activation can have many long-lasting effects in neurodevelopment.^1–6,32–34,41^ Therefore, the postnatal follow up of children exposed to SARS-CoV-2 prenatally, such as studies using MRI (https://www.kcl.ac.uk/research/bibs-brain-imaging-in-babies), is of utmost importance. Together, our findings suggest that early human foetal brain is vulnerable to SARS-CoV-2 infection and associated neuropathology, highlighting the importance of maternal and foetal monitoring during the COVID-19 pandemic.

## Supplementary Material

awac372_Supplementary_Data
