# Supplementary material for: Haemorrhage of human foetal cortex associated with SARS-CoV-2 infection
Source: Brain. 2023 Jan 16;146(3):1175–85. doi: 10.1093/brain/awac372 (PMC9976976; doi:10.1093/brain/awac372)

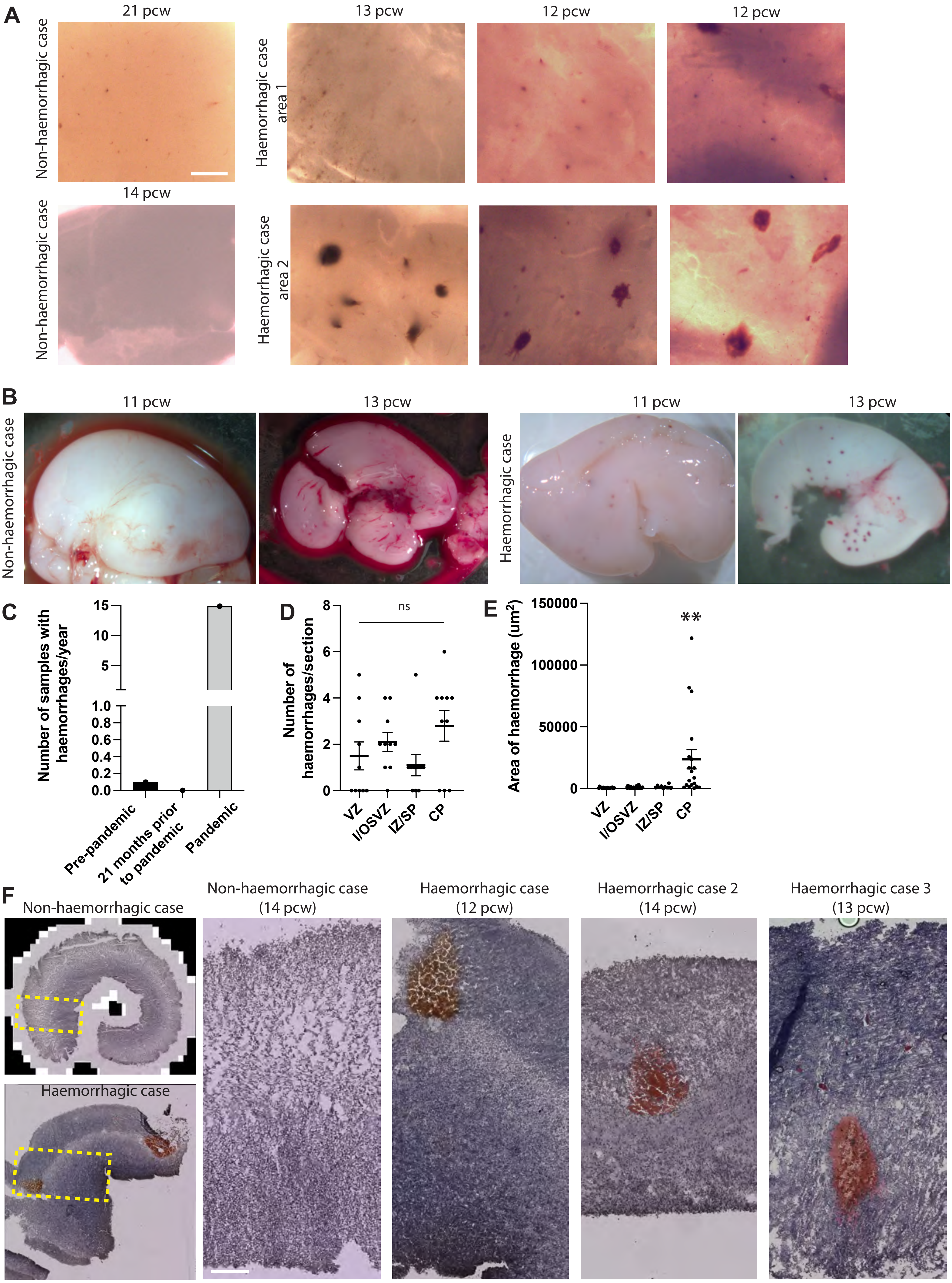

A
21 pcw
13 pcw
12 pcw
12 pcw
Non-haemorrhagic case
Haemorrhagic case area 1
14 pcw
Non-haemorrhagic case
Haemorrhagic case area 2
B
11 pcw
13 pcw
11 pcw
13 pcw
Non-haemorrhagic case
Haemorrhagic case
C
Number of samples with haemorrhages/year
15
10
5
1.0
0.8
0.6
0.4
0.2
0.0
Pre-pandemic
21 months prior to pandemic
Pandemic
D
Number of haemorrhages/section
ns
8
6
4
2
0
VZ
I/OSVZ
IZ/SP
CP
E
Area of haemorrhage (um²)
150000
100000
50000
0
**
VZ
I/OSVZ
IZ/SP
CP
F
Non-haemorrhagic case
Haemorrhagic case
Non-haemorrhagic case (14 pcw)
Haemorrhagic case (12 pcw)
Haemorrhagic case 2 (14 pcw)
Haemorrhagic case 3 (13 pcw)

Supplementary Figure 2. COVID-19 cases and numbers of human fetal cortex tissue samples.

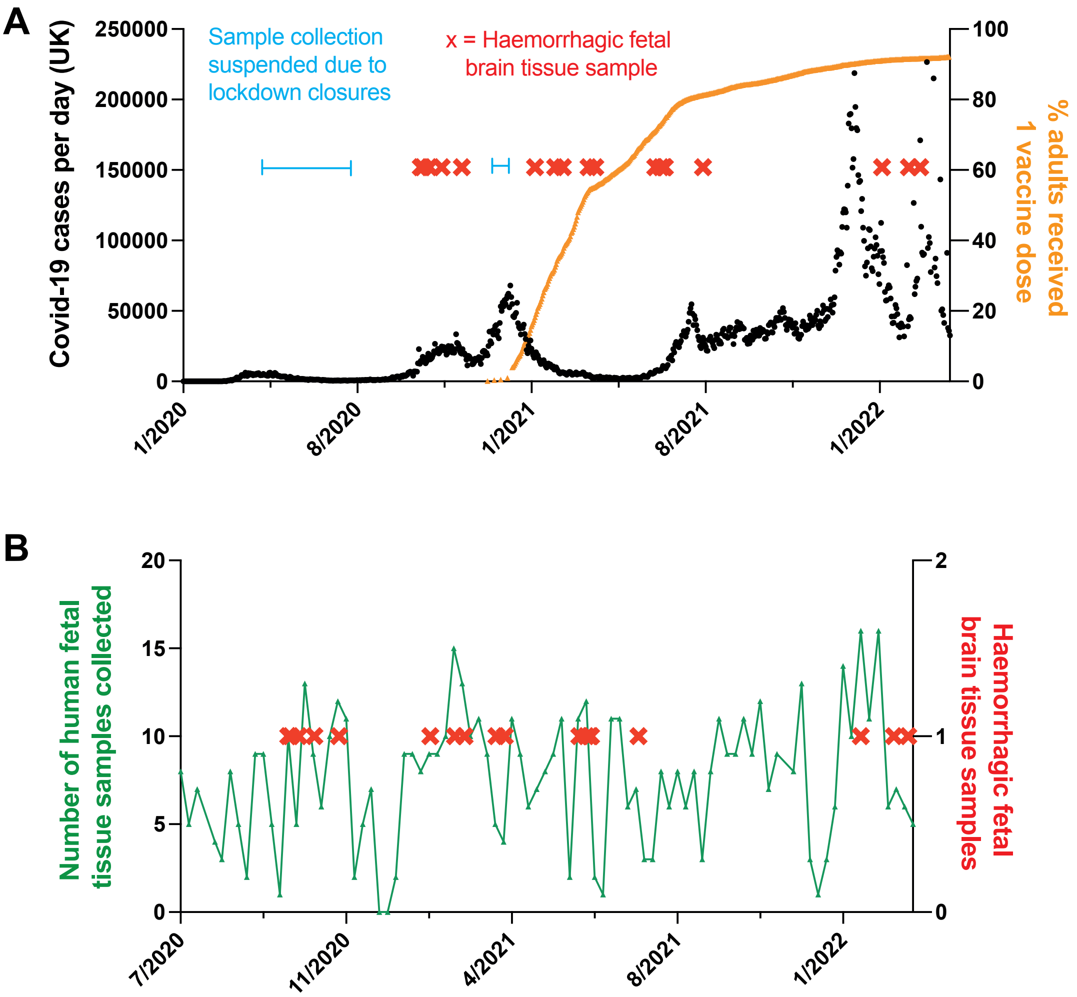

Supplementary Figure 3. Cleaved caspase-3 in fetal cortex tissue.

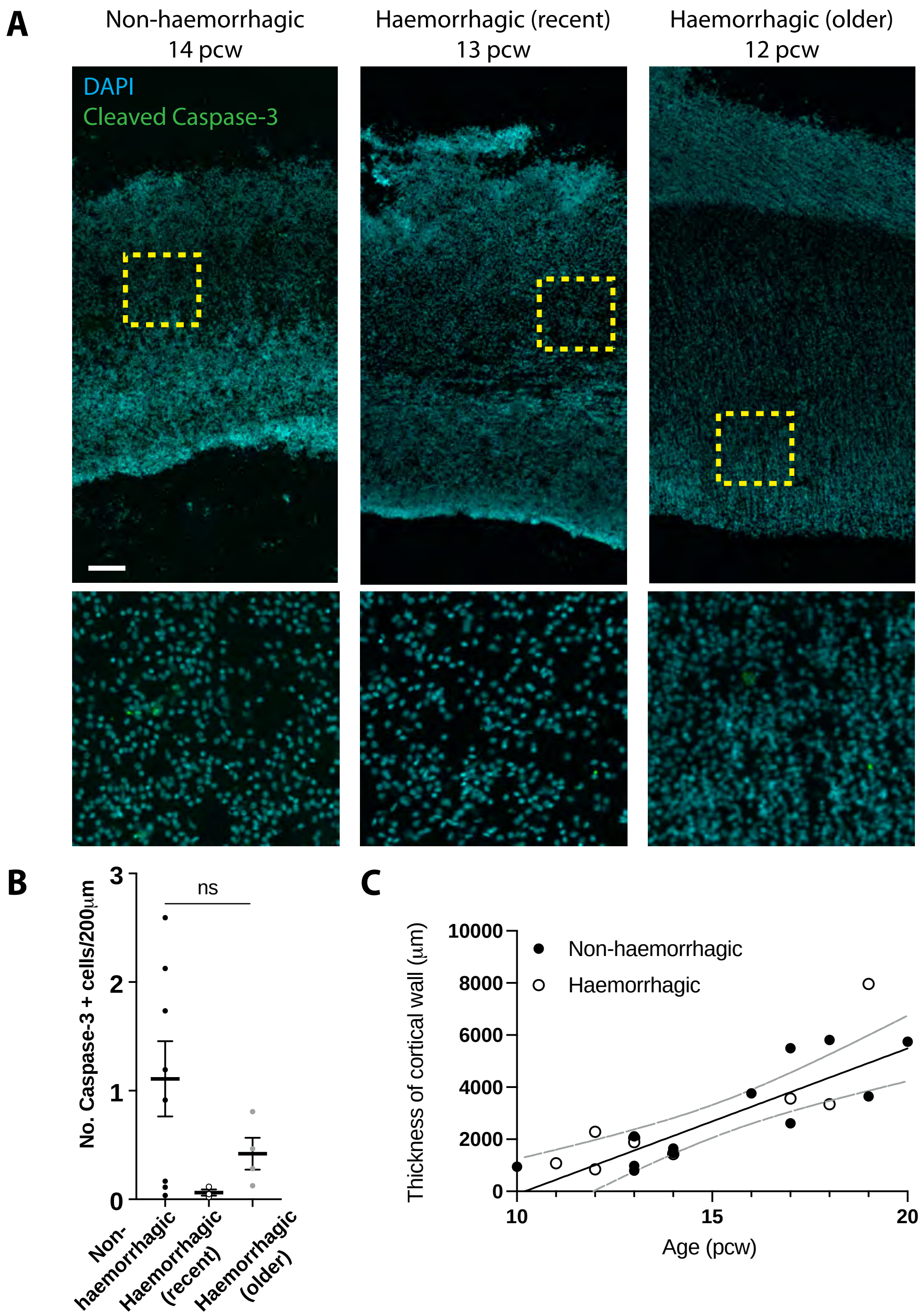

Supplementary Figure 4. SARS-CoV-2 spike protein in the choroid plexus.

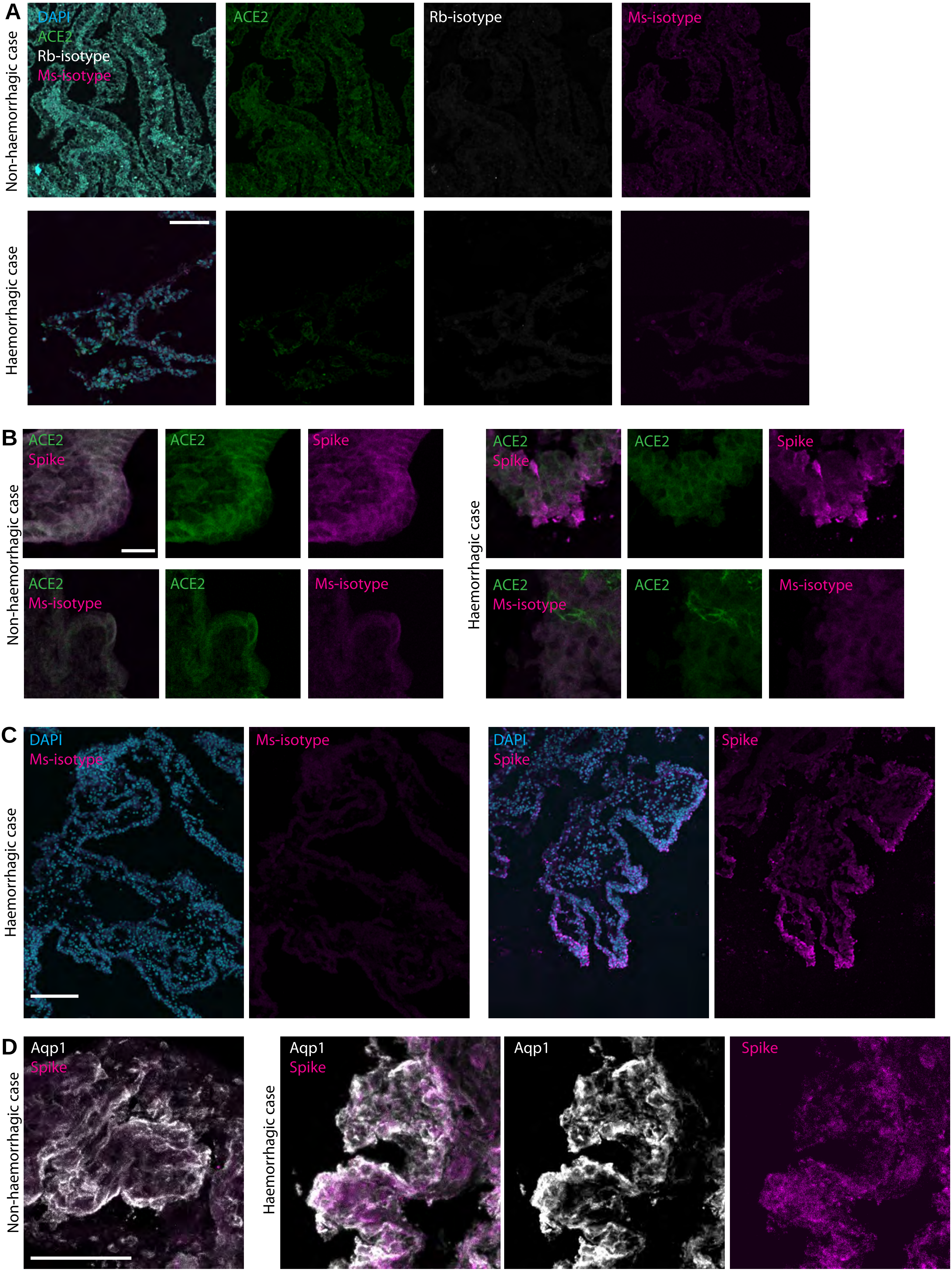

Supplementary Figure 5. SARS-CoV-2 spike protein in the cortex.

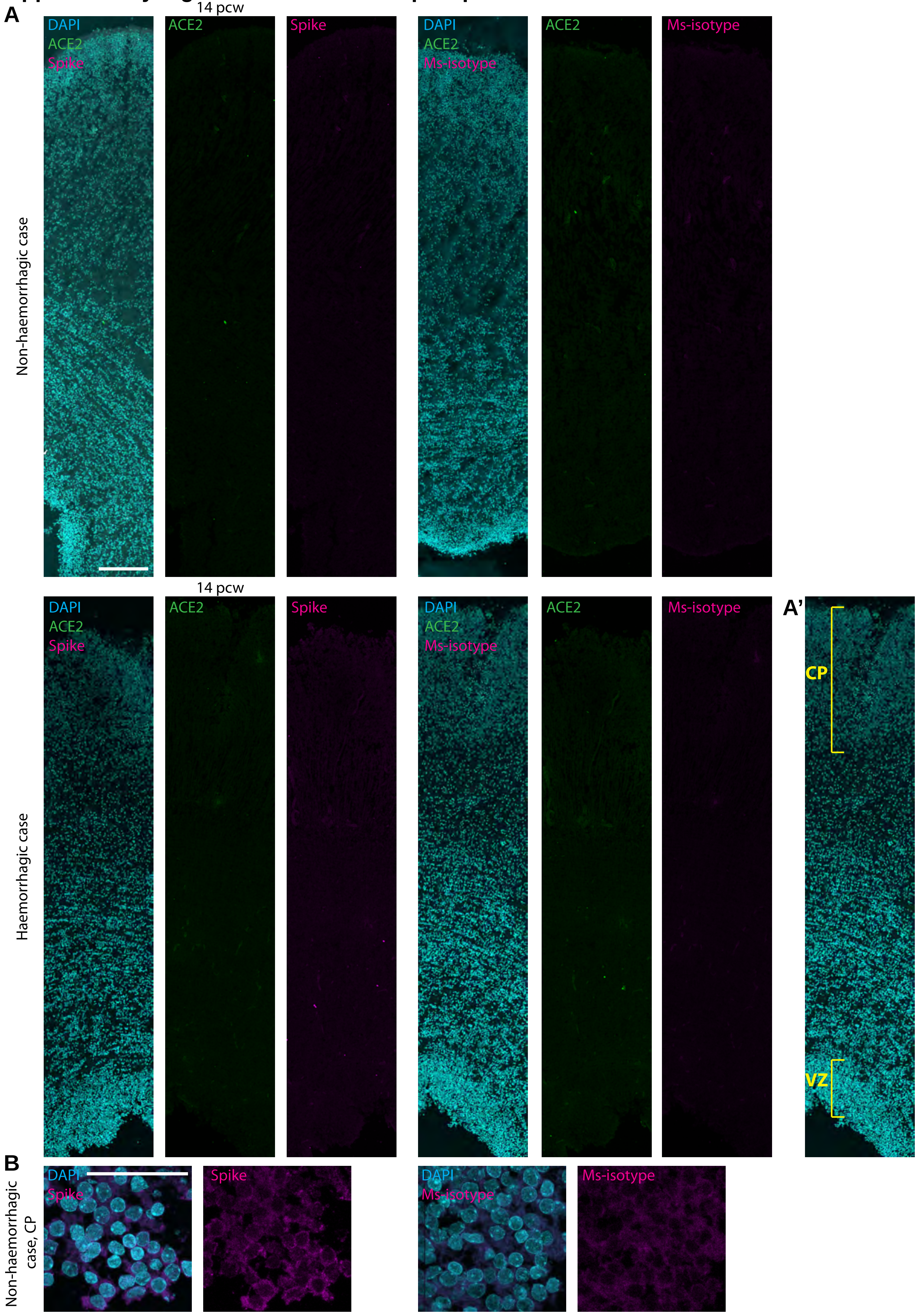

Supplementary Figure 6. SARS-CoV-2 spike protein in the cortex in haemorrhagic and pre-pandemic samples.

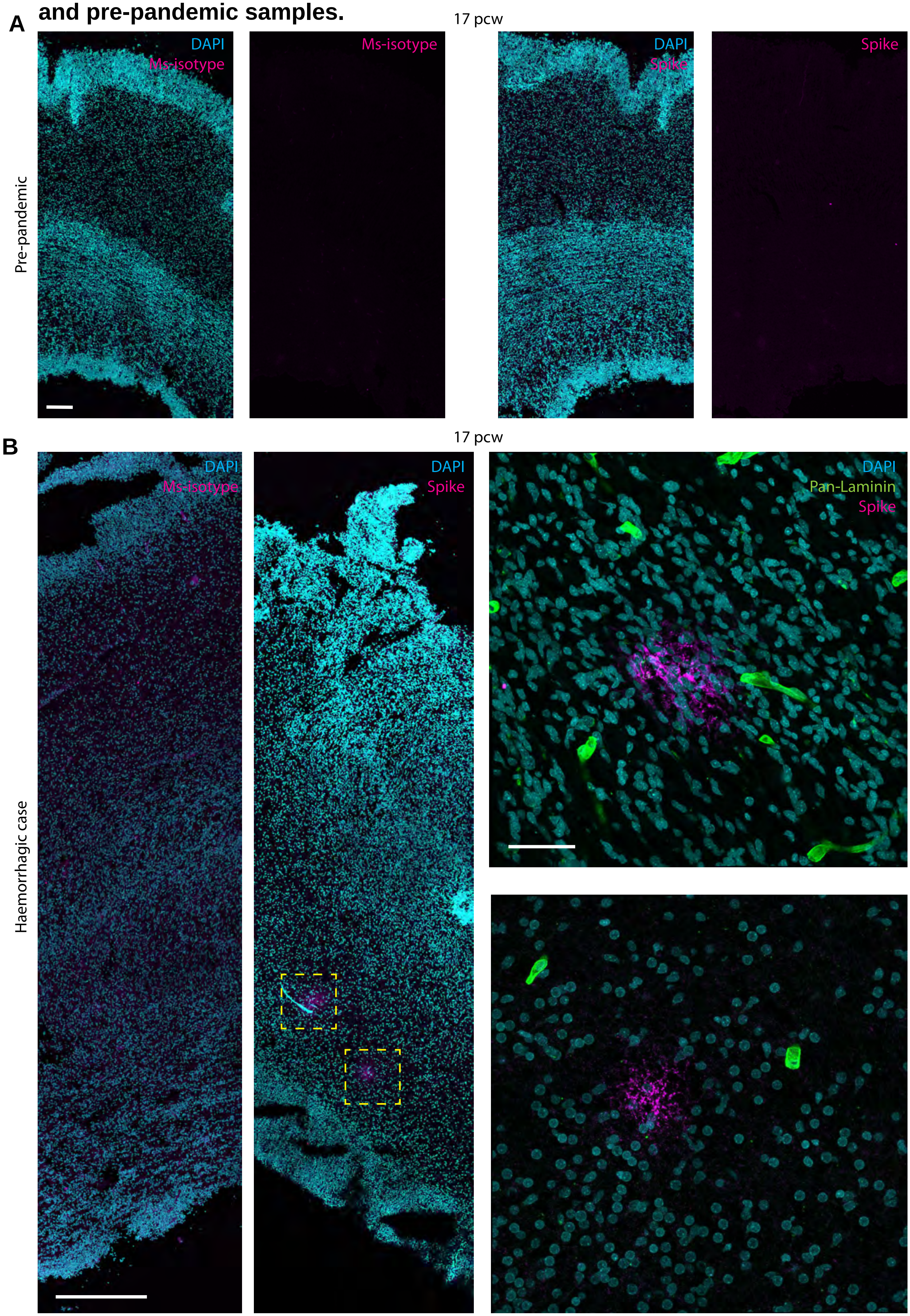

Supplementary Figure 7. SARS-CoV-2 spike protein and cell markers in haemorrhagic cortex.

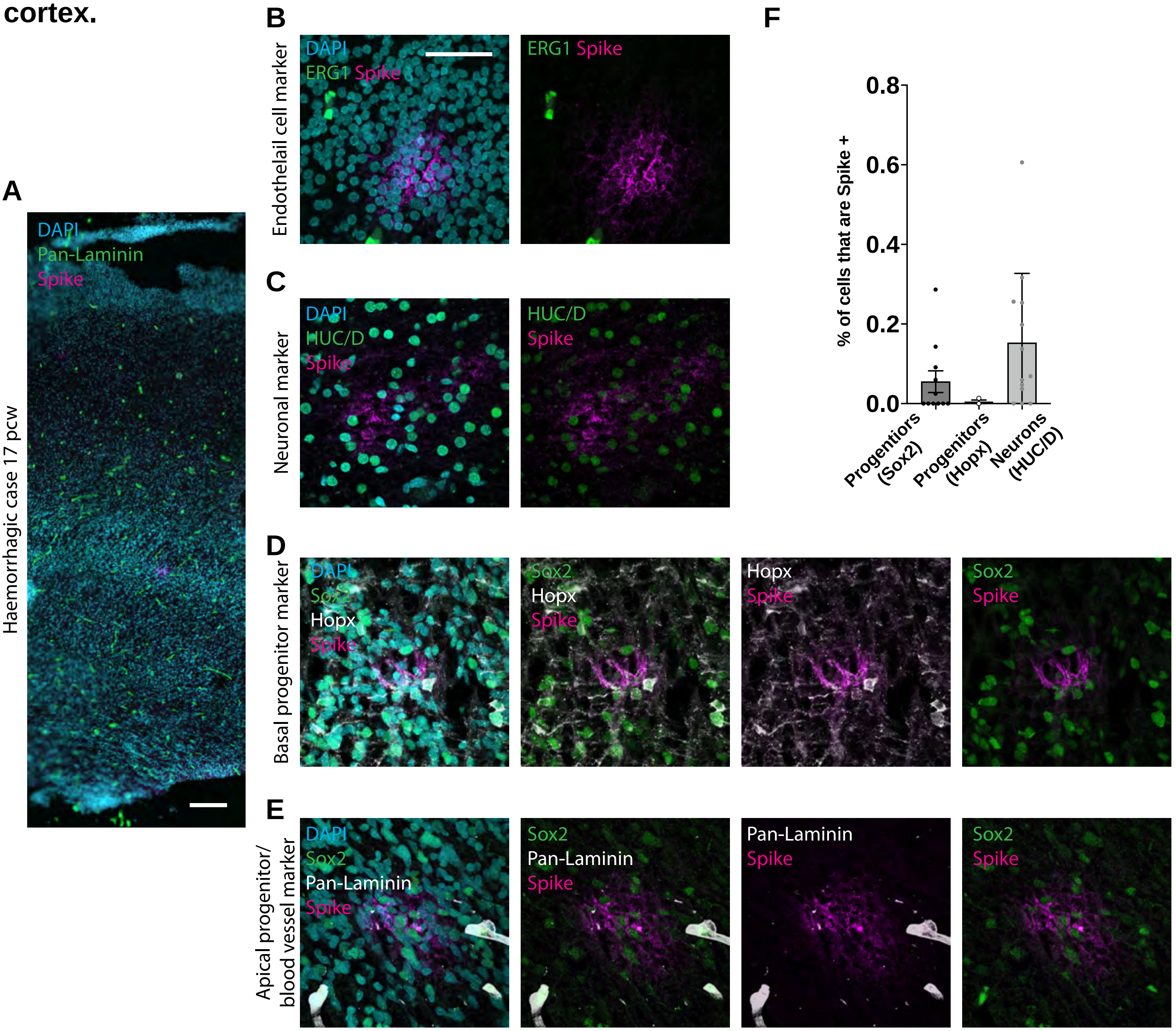

Supplementary Figure 8. Detection of SARS-CoV-2 in fetal cortex and COVID-19 patient lung tissue.

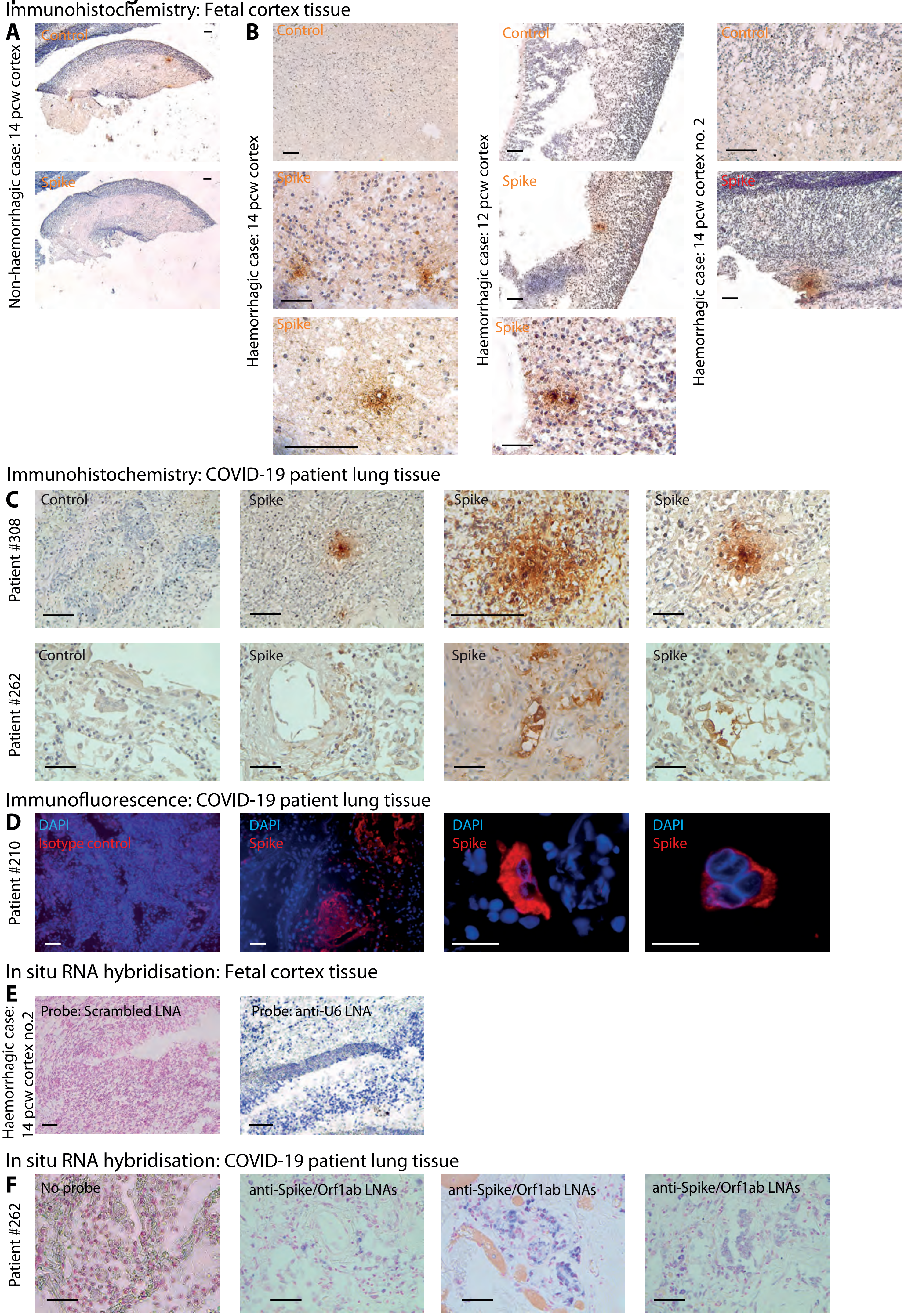

Supplementary Figure 9. SARS-CoV-2 spike protein in placenta, amnion and umbilical cord.

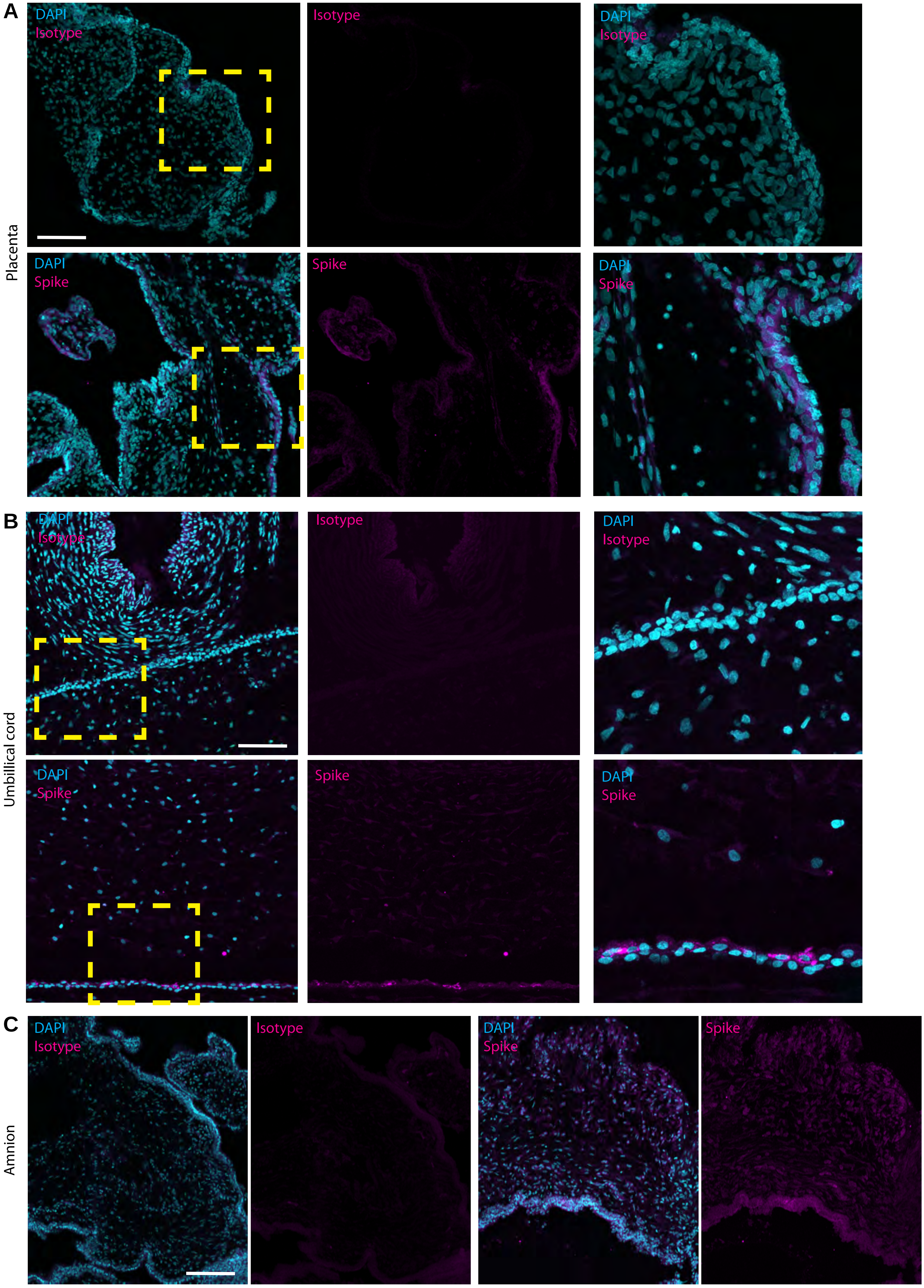

Supplemental Figure 10. Immune cell staining in fetal tissues.

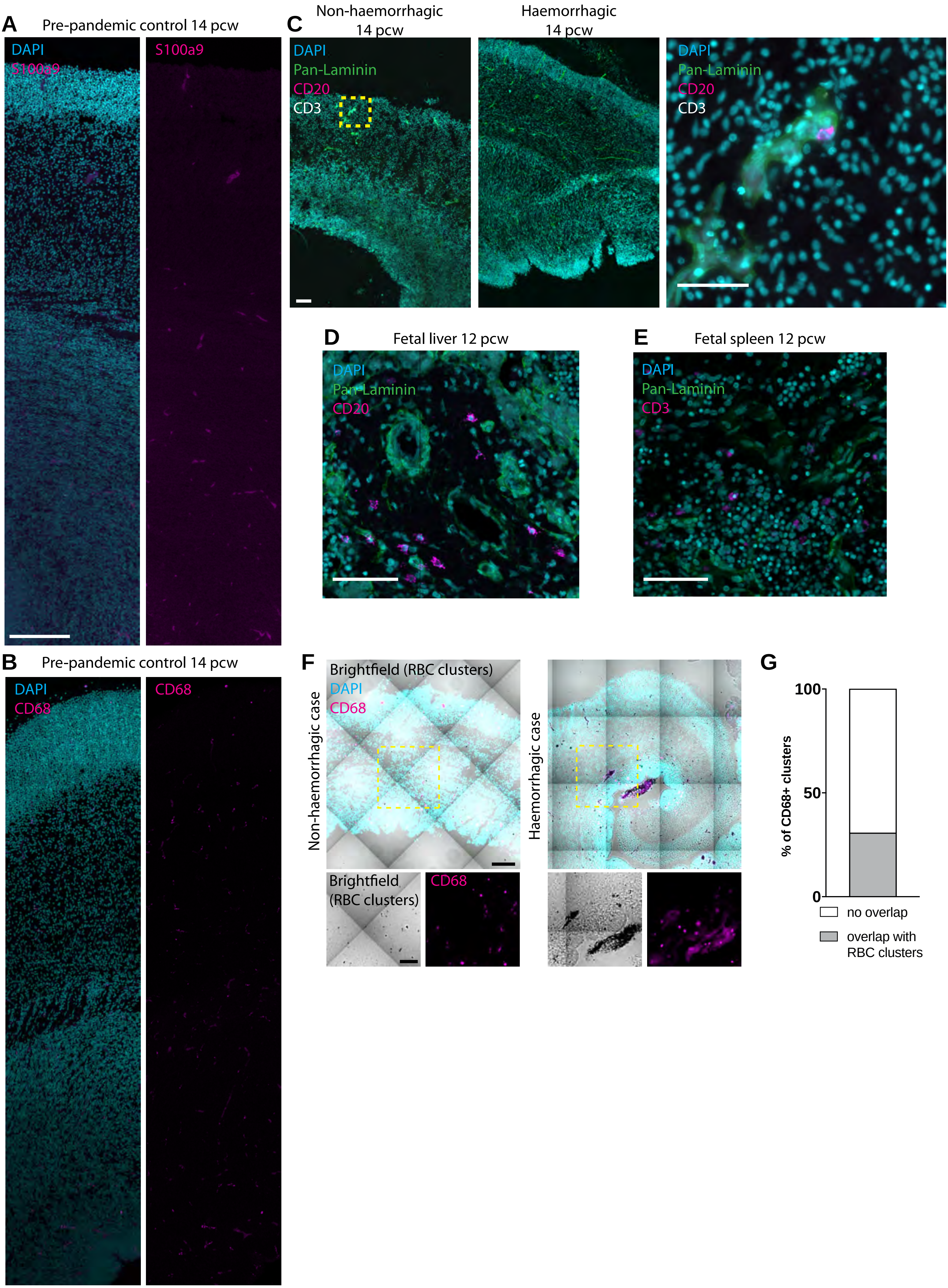

Supplement: awac372_Supplementary_Data [file awac372_supplementary_data.zip › brain-2022-00820-File006.pdf]
