# Supplementary material for: Haemorrhage of human foetal cortex associated with SARS-CoV-2 infection
Source: Brain. 2023 Jan 16;146(3):1175–85. doi: 10.1093/brain/awac372 (PMC9976976; doi:10.1093/brain/awac372)
Supplement: awac372_Supplementary_Data [file awac372_supplementary_data.zip › brain-2022-00820-File007.pdf]

Supplementary Figure legends

Supplementary Figure 1. Red blood cell clusters in human fetal cortex tissue.
(**A-B**) Whole mount images of human fetal cortex tissue displaying RBC clusters in haemorrhagic and non-haemorrhagic tissue (as labelled) collected during the COVID-19 pandemic (**A**) or prior to the COVID-19 pandemic (**B**). (**C**) Quantification of the yearly rate of haemorrhaged samples prior to the pandemic (<December 2019, 0.09 samples per year), the 21 months prior to the pandemic (May 2018 to February 2020, 0 samples per year) and during the pandemic (July 2020 to the middle of April 2022, 14.8 samples per year). (**D**, **E**) Quantification of the number (**D**) and area (**E**) of haemorrhages in the ventricular zone (VZ), inner and outer subventricular zones (I/OSVZ), intermediate zone/subplate (IZ/SP) and cortical plate (CP). Error bars = SD, p = ns=0.3713, *=<0.009. n=10. (**F**) H&E staining of haemorrhagic and non-haemorrhagic tissue. Corresponding whole mount tissue images can be found in Fig. 1A (haemorrhagic 12 pcw) and Fig. 1B (haemorrhagic 12 pcw, 14pcw) and panels in A of this figure (haemorrhagic 13 pcw and non- haemorrhagic 14 pcw). Yellow boxes delineate the areas shown. Scale bars 1 mm (**A**, **B**), 200 µm (**F**).

Supplementary Figure 2. COVID-19 cases and numbers of human fetal cortex tissue samples.
Graphs showing the number of confirmed COVID-19 cases per day in the UK from 30th January 2020 up to 14th April 2022 (black), the haemorrhagic fetal cortex samples (red crosses) and (**A**) the percentage of the adult population that have received one dose of vaccine (yellow line), and (**B**) the numbers of total human fetal tissue samples collected during the same period (green line).

Supplementary Figure 3. Cleaved caspase-3 in fetal cortex tissue.
(**A**) Immunofluorescence for cleaved caspase-3 (green) and DAPI (blue) in cortex from non-haemorrhagic (left) and haemorrhagic (centre, right) samples. The haemorrhagic samples contain either mostly recent haemorrhages (centre, as presented in Fig. 1H) or older haemorrhages (right, as presented in Fig. 1H). Yellow boxes delineate the areas shown below. Scale bar 200 µm. (**B**) Quantification of the number of cleaved caspase-3 positive cells per 200 µm wide column of

cortical tissue. Error bars = SD. Non-haemorrhagic samples n=8, haemorrhagic (recent) n=3, haemorrhagic (older) n=4. One-way ANOVA, p = ns=0.14571. (**C**) Quantification of the thickness of the cortical wall for non-haemorrhagic (black) and haemorrhagic (white) samples. Black line indicates line of best fit for non-haemorrhagic samples, grey lines show 95% confidence intervals. T=test, p = ns=0.7492.

Supplementary Figure 4. SARS-CoV-2 spike protein in the choroid plexus.
(**A**) Immunofluorescence of choroid plexus from fetuses with non-haemorrhagic (upper) and haemorrhagic (lower) cortex, for ACE2 (green), rabbit isotype control (for SARS-CoV-2 nucleocapsid, grey) and mouse isotype control (for SARS-CoV-2 spike protein, magenta) and their merge with DAPI (blue). (**B**) Immunofluorescence of choroid plexus from fetuses with non-haemorrhagic (left) and haemorrhagic (right) cortex, for ACE2 (green) and either spike protein (upper, magenta) or mouse isotype control (lower, magenta). (**C**) Immunofluorescence of choroid plexus from a fetus with a haemorrhagic cortex, for either spike protein (left, magenta) or mouse isotype control (right, magenta). (**D**) Immunofluorescence of choroid plexus from fetuses with non-haemorrhagic (left) and haemorrhagic (right) cortex, for aquaporin-1 (grey) and SARS-CoV-2 spike protein (magenta). Scale bars (**A**) 100 µm, (**B**) 25 µm, (**C**) 200 µm and (**D**) 50 µm.

Supplementary Figure 5. SARS-CoV-2 spike protein in the cortex.
(**A**) Immunofluorescence of cortex from non-haemorrhagic (upper) and haemorrhagic (lower) fetuses for ACE2 (green) and either spike protein (left, magenta) or mouse isotype control (for SARS-CoV-2 spike protein, right, magenta) and their merge with DAPI (blue). (**A'**) DAPI image from neighbouring panel (**A**, haemorrhagic, right panels) with the cortical plate (CP) and ventricular zone (VZ) indicated in yellow. (**B**) Immunofluorescence of cortex from non-haemorrhagic fetuses for either spike protein (left, magenta) or mouse isotype control (for SARS-CoV-2 spike protein, right, magenta) and their merge with DAPI (blue). Scale bars 200 µm (**A**) and 50 µm (**B**).

Supplementary Figure 6. SARS-CoV-2 spike protein in the cortex in haemorrhagic and pre-pandemic samples.

(**A**) Immunofluorescence of cortex from a pre-pandemic fetuses for either spike protein (right, magenta) or mouse isotype control (for SARS-CoV-2 spike protein, left, magenta) and their merge with DAPI (blue). (**B**) Immunofluorescence of cortex from a haemorrhagic fetus for either spike protein (right, magenta) or mouse isotype control (for SARS-CoV-2 spike protein, left, magenta) and their merge with DAPI (blue). Yellow boxes delineate the areas shown to the right, with spike protein (magenta), pan-laminin (green) and DAPI (blue). Scale bars 200 µm (**A**) and 500 µm (**B**) and 50 µm (inset **B**).

Supplementary Figure 7. SARS-CoV-2 spike protein and cell markers in haemorrhagic cortex.
(**A-E**) Immunofluorescence for SARS-CoV-2 spike protein (magenta) and DAPI (blue) in fetal cortex from a haemorrhagic sample with either pan-laminin (green, **A**), ERG (endothelial cell marker, green, **B**), HuC/D (neuronal marker, green, **C**), sox2 (progenitor marker, green) and hopx (basal progenitor maker, grey) (**D**), or sox2 (green) and pan-laminin (grey) (**E**). (**F**) Quantification of the percentage of cells positive for SARS-CoV-2 spike protein: mean sox2 0.055%, hopx 0.004%, HuC/D 0.152%. Scale bars 200 µm (**A**) and 50 µm (**B**).

Supplementary Figure 8. Detection of SARS-CoV-2 in fetal cortex and COVID-19 patient lung tissue.
(**A-C**) Immunohistochemistry for SARS-CoV-2 spike protein (Spike) or mouse isotype control (Control) of fetal cortex tissue from non-haemorrhagic (**A**) and haemorrhagic (**B**) samples, and of COVID-19 patient lung tissue (**C**). (**D**) Immunofluorescence for SARS-CoV-2 spike protein in COVID-19 patient lung tissue. (**E**, **F**) In situ hybridisation of fetal cortex tissue from a haemorrhagic sample (**E**) and of COVID-19 patient lung tissue (**F**) for scrambled LNA (left panel, **E**), no probe (left panel, **F**) or anti-U6 LNA (right panels, **E**, **F**) controls. Scale bars 100 µm (**A-C**, **D** left panels, **E**, **F**) and 10 µm (**D**, right panels).

Supplementary Figure 9. SARS-CoV-2 spike protein in placenta, amnion and umbilical cord.
(**A-C**) Immunofluorescence for either spike protein (lower, magenta) or mouse isotype control (for SARS-CoV-2 spike protein, upper, magenta) and their merge with DAPI (blue) of placenta

from an 11 pcw haemorrhagic sample (**A**), umbilical cord from a 13 pcw sample (**B**) and amnion from a 9pcw haemorrhagic sample (**C**). Yellow boxes delineate the areas shown to the right. Scale bars 100 µm.

Supplemental Figure 10. Immune cell staining in fetal tissues.

(**A**, **B**) Immunofluorescence of pre-pandemic fetal cortex tissue for S100a9 (**A**, magenta) or CD68 (**B**, magenta) and their merge with DAPI (blue). Scale bar 200 µm. (**C**) Immunofluorescence of cortex from non-haemorrhagic and haemorrhagic samples for pan-laminin (green), lymphocyte markers CD20 (magenta) and CD3 (grey) and DAPI (blue). Yellow boxes delineate the areas shown to the right, with a scale bar of 50 µm. (**D**, **E**) Immunofluorescence for pan-laminin (green) CD20 (**D**, magenta) and CD3 (**E**, magenta) staining in fetal liver (**D**) and spleen (**E**). Scale bar 50 µm. (**F**) Brightfield and immunofluorescence for DAPI (blue) and CD68 (magenta) of cortex from non-haemorrhagic (left) and haemorrhagic (right) fetuses. Yellow boxes delineates areas below showing RBC clusters (brightfield) and CD68 cell clusters (magenta). Scale bars 200 µm, inset 100 µm. (**G**) Quantification of the percentage of CD68 positive clusters that overlap (grey) with a RBC cluster.
